# Interleukin-23 may contribute to the pathogenesis of lumbar disc herniation through the IL-23/IL-17 pathway

**DOI:** 10.1186/s13018-016-0343-8

**Published:** 2016-01-16

**Authors:** Hongqiang Jiang, Yao Deng, Tao Wang, Jianxiong Ma, Pengfei Li, Peng Tian, Chao Han, Xinlong Ma

**Affiliations:** Department of Orthopaedics, Tianjin Medical University General Hospital, No. 154, Anshan Road, Heping District, Tianjin, 300052 China; Tianjin Orthopedics Institute, No.155, Munan Road,Heping District, Tianjin, 300050 China

**Keywords:** Lumbar disc herniation, Intervertebral disc, IL-23, IL-17

## Abstract

**Background:**

Studies have indicated that interleukin 23 (IL-23) plays an important role in many inflammatory- and autoimmune-related diseases. However, there is little knowledge about IL-23 in lumbar disc herniation (LDH). Thus, in this study, we aimed to find out whether IL-23 is expressed in intervertebral discs (IVDs) and what roles it may play.

**Methods:**

Human IVD tissues were collected from 29 LDH patients and 8 vertebral fracture patients (normal control, NC group). According to the integrity of annulus fibrosus, LDH patients were divided into two groups: R group (ruptured group, *n* = 16) and NR group (non-ruptured group, *n* = 13). Morphological changes of IVDs were assessed by hematoxylin and eosin (HE staining), and expression of IL-23 in IVD tissues was detected by immunohistochemical staining. Besides gene expression of IL-23, IL-17, IL-6, IL-1β, and TNF-α was also evaluated by reverse transcription polymerase chain reaction (RT-PCR).

**Results:**

The results showed that the R group was more degenerated than the other two groups and NC group showed the least degenerated performance; stronger positive IL-23 expression was observed in herniated IVDs, especially in the R group. Meanwhile, higher gene expression of IL-23, IL-17, IL-6, IL-1β, and TNF-α was found in the tissues from LDH patients and a positive correlation between IL-17 and IL-23 gene expression was also observed.

**Conclusions:**

Taken all above results together, it may be deduced that higher expression of IL-23 may contribute to the deterioration of IVDs through the IL-23/IL-17 pathway.

## Background

Lumbosacral radiculopathy is a common and costly health problem, which often occurs as a result of mechanical compression and biochemical irritation on the nerve root by lumbar disc herniation (LDH) [1]. Although mechanical deformation may contribute to the radicular pain, it is currently believed that autoimmune reaction and related inflammatory mediators are the key players in causing this symptom. Nucleus pulposus (NP) was believed to be an autoantigen; this is supported by accumulation of lymphocytes in local disc tissues following exposure to autologous NP or injury to the annulus fibrosus [2]. some animal studies proved that inflammatory and immune response could be induced by a healthy autologous NP which was applied to nerve root, as a model of non-compressive disc herniation, resulting in neuronoglial apoptosis, a decrease in nerve conduction velocity, the onset of gait abnormality, mechanical allodynia and thermal hyperalgesia [3–9]. Inflammatory cells (mainly macrophages) from the intervertebral disc (IVD) tissues can secrete many pro-inflammatory mediators and regulatory cytokines, such as prostaglandin E2 [10–12], TNF-α [13–18], IL-1β [13, 14], IL-6 [13, 19], IL-8 [20], IL-12 [19, 21], nerve growth factor [22], vascular endothelial growth factor [23], and substance P [22, 24].

Interleukin 23 (IL-23) is a heterodimeric pro-inflammatory cytokine, which is mainly secreted by activated macrophages. It can help Th17 cells to expand and maintain their lineage [25, 26], meanwhile promoting the production of IL-17 without the engagement of T cell receptor in γσT cells [27]. Thus, both IL-23 and IL-17 form a new axis through Th17 cells, which play an important role in many autoimmunity and chronic inflammation diseases, such as experimental autoimmune encephalomyelitis, rheumatoid arthritis, and inflammatory bowel disease [28–30], and targeting on them has become a new strategy for these kind of diseases as some exciting news have been reported in several articles [31, 32].

Recently, studies have proven that Th17 cells and IL-17 was infiltrated and expressed in IVD tissues and may contribute a lot to the local inflammation and radicular pain [33–36]. Take into consideration that macrophages are the predominantly infiltrated cells in herniated discs and can secrete IL-23 when they are activated, we may speculate that IL-23/Th17/IL-17 axis may still have a contribution to the pathogenesis of LDH. However, there is little knowledge about IL-23 in LDH up to now. Thus, the current study was carried out to investigate whether IL-23 was expressed in IVD tissues and whether IL-23/IL-17 pathway plays a role in the pathogenesis of LDH.

## Methods

### Source of human IVD tissues

From August 2014 to January 2015, 29 patients diagnosed with LDH were randomly recruited from the Spine Department of Tianjin Hospital. The enrolled patients should meet all the following criteria: (1) typical osphyalgia or sciatica symptoms, (2) positive Lasegue’s sign or Bragard test, (3) single lumbar disc herniation, and (4) symptoms persist or recur repeatedly, and conservative treatments were failed. In addition, eight persons who had lumbar fusion surgery because of lumbar vertebral fracture were also included in the experiment and used as normal controls. Individuals with one of the following circumstances should be excluded: (1) history of osteoarthritis, spondylosis, spondylolisthesis, acute or chronic inflammatory disease, hypertension, or diabetes mellitus; (2) have taken or are currently taking immunosuppressive drugs; and (3) previous surgery for IVD disease.

Patients with LDH can be divided into two groups by using the method described by Cheng et al [33]. In short, patients in whom the annulus fibrosus was intact and the nucleus pulposus was completely sealed by the annulus fibrosus were categorized as non-ruptured group (NR, 13 patients). Patients in whom the annulus fibrosus was ruptured and the nucleus pulposus was not completely sealed by the annulus fibrosus and thus the nucleus pulposus was exposed to circulation were categorized as ruptured group (R, 16 patients). For a diagnosis of rupture, both MRI evidence of rupture and visual observation of rupture during surgery had to be present.

During surgery, after removal of the intervertebral disc, it was immediately washed by phosphate-buffered solution (PBS) for three times to clean the remained blood. Subsequently, the NP was separated from the AF by using a stereotaxic microscope and then the NP was cut into two parts: one was fixed in 10 % neutral formalin and used for morphological observation, and the other was placed in a cryopreservation tube and preserved at −80 °C for reverse transcription polymerase chain reaction (RT-PCR). The characteristics of the enrolled subjects are shown in Table 1.

**Table 1:** Characteristics of the enrolled subjects

Case no.	Gender	Diagnosis	Age (years)	Level	HE	IHC	RT-PCR
1	F	NC	23	L2	Y	Y	Y
2	M	NC	36	L4	Y	Y	Y
3	M	NC	58	L1	Y	Y	Y
4	M	NC	42	L3	Y	Y	Y
5	F	NC	37	L3	Y	Y	Y
6	M	NC	42	L4	Y	Y	Y
7	M	NC	32	L3	Y	Y	Y
8	M	NC	34	L3	Y	Y	Y
9	F	NR	43	L4/5	Y	Y	Y
10	M	NR	31	L4/5	Y	Y	Y
11	M	NR	39	L5/S1	Y	Y	Y
12	M	NR	42	L4/5	Y	Y	Y
13	F	NR	44	L4/5	Y	Y	N
14	F	NR	48	L4/5	Y	Y	Y
15	M	NR	67	L5/S1	Y	Y	Y
16	M	NR	51	L5/S1	Y	Y	Y
17	F	NR	50	L5/S1	Y	Y	Y
18	M	NR	42	L3/L4	Y	Y	N
19	F	NR	47	L5/S1	Y	Y	Y
20	M	NR	53	L5/S1	Y	Y	Y
21	M	NR	41	L4/5	Y	Y	Y
22	M	R	45	L5/S1	Y	Y	Y
23	F	R	52	L5/S1	Y	Y	Y
24	M	R	43	L4/5	Y	Y	Y
25	F	R	30	L4/5	Y	Y	Y
26	F	R	41	L5/S1	Y	Y	Y
27	M	R	47	L4/5	Y	Y	Y
28	M	R	57	L5/S1	Y	Y	N
29	F	R	41	L5/S1	Y	Y	Y
30	M	R	53	L5/S1	Y	Y	N
31	M	R	52	L5/S1	Y	Y	Y
32	M	R	49	L5/S1	Y	Y	Y
33	F	R	56	L4/5	Y	Y	Y
34	M	R	64	L5/S1	Y	Y	N
35	F	R	39	L4/5	Y	Y	Y
36	M	R	42	L4/5	Y	Y	Y
37	F	R	57	L5/S1	Y	Y	Y

*F* female, *M* male, *NC* normal control group, *NR* non-ruptured group, *R* ruptured group, *Y* yes, *N* no

### HE staining

IVD tissues obtained from the surgery were fixed in 10 % neutral formalin for 24 h. After that, they were dehydrated in alcohol and transparentized in xylene. Then, they were embedded in paraffin wax and cut into 5-μm sections. Subsequently, the sections were dewaxed to water and stained by hematoxylin and eosin (HE) and sealed with neutral gum. The pathological changes of the intervertebral disc were evaluated by using a light microscope (Nikon, Japan).

### Immunohistochemical localization of IL-23

IVD specimens obtained from LDH and vertebral fractures were embedded in paraffin, and sections were cut at 4 μm and mounted on slides and dried at 60 °C. Sections were deparaffinized in xylene and rehydrated through graded alcohols to distilled water. Then, the sections were incubated with H_2_O_2_ for 10 min to eliminate the activity of endogenous peroxidase, followed by incubation for 2.5 h with human IL-23 immunogen affinity purified polyclonal antibody (Abcam, ab115759) diluted 1:200 in blocking buffer. The sections were washed with PBS and incubated for 30 min with HRP-labeled goat anti-rabbit IgG secondary antibodies (Fitzgerald, 43R-1614, USA) in blocking buffer (1:1000). Color was developed with diaminobenzidine, and the sections were counterstained with hematoxylin for 1 min at room temperature to stain the cell nuclei. Sections were imaged by using a microscope (Nikon, Japan) with ×20 and ×40 objective lenses. Human kidney tissue was used as the positive control.

Semiquantitative grading of IL-23 immunoreactivity in immunostained sections was performed by two graders who evaluated eight separate ×20 magnification fields for each tissue sample. The method was used as previously described by Shamji et al [19]. And this strategy can provide the most complete and comprehensive evaluation of the surgical tissue samples.

Scores were given for degree of cytokine immunoreactivity as follows: 0 = no positive cells and 1 = at least one positively labeled cell.

### RT-PCR

As few annulus fibrosus was found in the dissected intervertebral disc tissues in our experiment, and nucleus pulposus was generally believed to play a central role in the pathogenesis of LDH, thus only the nucleus pulposus was prepared for the RNA extraction. Tissue samples were grinded in liquid nitrogen and homogenized in 1 ml TRIzol ®Reagent (Invitrogen, Carlsbad, CA, USA) per 100 mg of tissue. The purity and concentration of the extracted total RNA were evaluated by an ultraviolet spectrophotometer (Thermo Fisher NanoDrop-1000, USA). According to the manufacturer’s protocol, 1 μg of total RNA was used to synthesize cDNA using ReverTra Ace qRCR RT Kit (Toyobo, Osaka, Japan). Real-time PCR amplifications were performed using gene-specific primers in a final concentration about 0.4 μM and SYBR® Green Realtime PCR Master Mix (TOYOBO, OSAKA, JAPAN) according to the manufacturer’s protocol. The primer sequences used this in this experiment are shown in Table 2. The thermal cycling conditions were as follows: an initial denaturation at 95 °C for 1 min, followed by 40 cycles of 10 s of denaturing at 95 °C, 15 s of annealing at 58 °C, and 20 s of extension at 72 °C. The expression levels of the target genes were normalized to that of β-actin in the same cDNA samples.

**Table 2:** Sequences of primers for the RT-PCR assays

Gene	Sense	Sequence 5′–3′
IL17A	F	CCATAGTGAAGGCAGGAATC
	R	GAGGTGGATCGGTTGTAGTA
IL23A	F	GACACATGGATCTAAGAGAAGAG
	R	AACTGACTGTTGTCCCTGAG
IL-1β	F	TGTTGAAAGATGATAAGCCCACTCT
	R	CAAATCGCTTTTCCATCTTCTTC
IL-6	F	CGGGAACGAAAGAGAAGCTCTA
	R	GAGCAGCCCCAGGGAGAA
TNF-α	F	CGAGTCTGGGCAGGTCTACTTT
	R	AAGCTGTAGGCCCCAGTGAGTT
β-actin	F	GCAGAAGGAGATCACTGCCCT
	R	GCTGATCCACATCTGCTGGAA

### Statistical analysis

All tests were performed by using SPSS 19.0. Data were expressed as the mean ± standard deviation (SD). Differences in immunoreactivity score between study groups were determined by using chi-squared test, and differences of cytokines expression levels between study groups were determined by using one-way analysis of variance (ANOVA). Pearson correlation coefficient was used to determine the relationship between IL-23 and IL-17. A two-tailed *p* < 0.05 was considered statistically significant.

## Results

### HE staining results

**Fig. 1:**
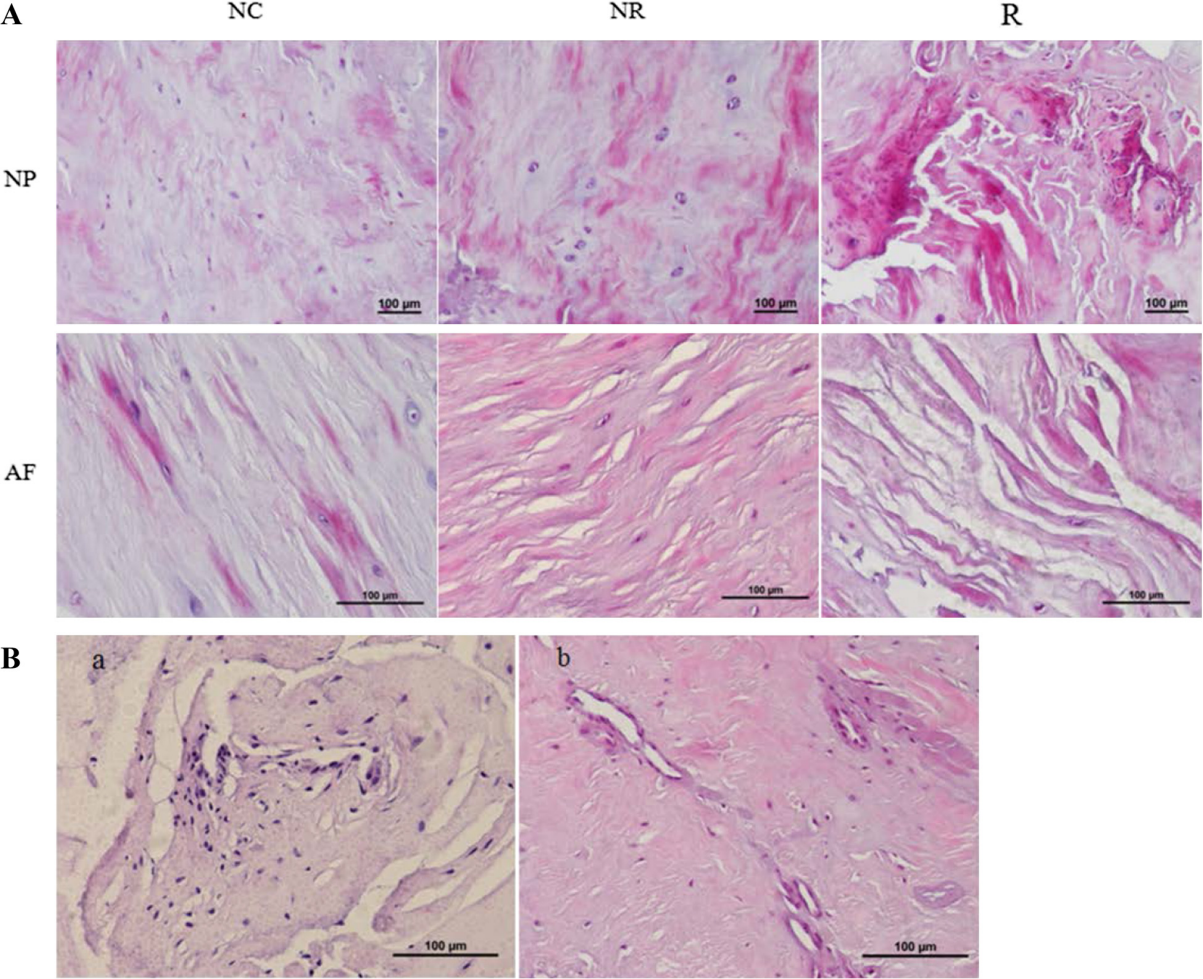
Morphological changes of lumbar intervertebral discs. **a** H&E staining of intervertebral disc tissues from the three groups. *R* ruptured group showed the most severe degenerative changes in the NP and AF; there are more degenerative performances in the *NR* non-ruptured group than the *NC* normal control group. Magnification: *NP* nucleus pulpous, in the *upper panel*, ×100; *AF* annulus fibrosus, in the *lower panel*, ×200. **b** Infiltrated lymphocytes (*a*) and small vessels (*b*) can be seen in the IVD tissues from the ruptured group. All magnifications were ×200

The morphology of the intervertebral disc was observed by using a light microscope (Nikon, Japan). Figure 1a showed the representative results of the three groups. According to the histological performance, we can see that IVD tissues from the ruptured group showed more severely degenerative performance than the other two groups, and there were less degenerative changes in the normal group than the non-ruptured group. To be more specific, in the normal control group, the structures of NP and AF were almost normal, and there were less cracks and small cell clusters in this group; however, in the non-ruptured group, the degenerative changes were more severe, cracks can be seen in this group samples, and there were more small cell clusters in the IVD tissues which is one of the features of degeneration. In the ruptured group, the structures of NP and AF were nearly destroyed, more cracks and fissures can be seen in this group, and it was not rare to see the performances of fibrinoid necrosis, small vessels, and lymphocytes infiltration (Fig. 1b).

### Immunohistochemical-staining of IL-23 in IVD tissues

**Fig. 2:**
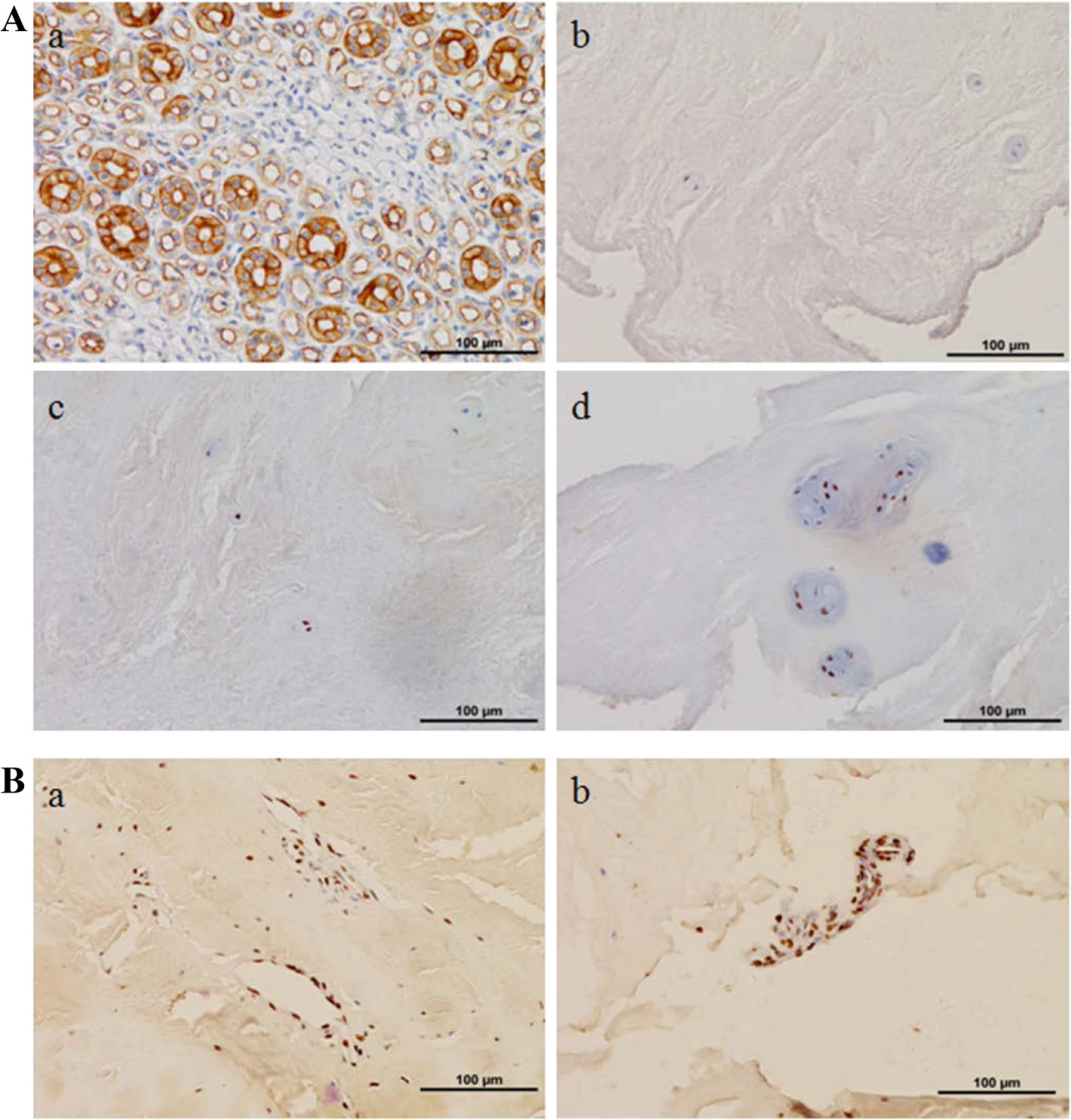
Representative results of IL-23 among different groups. All magnifications ×200. **a** (*a*) Human kidney tissues from the positive control. (*b*) IVD tissues from the normal control group. Nearly no positive cells can be recognized. (*c*) IVD tissues from the non-ruptured group. Only a few cells showed positive results. (*d*) IVD tissues from the ruptured group. Much higher positive results can be noted in this group than the other two groups. **b** In both the ruptured and non-ruptured groups, significant positive results of IL-23 can be found around the small vessels (*a*) and the infiltrated inflammatory cells (*b*)

Representative pictures and statistical results of the immunohistological staining of IL-23 in the IVD specimens were shown in Table 3 and Fig. 2a. We can see that there were nearly no positive expressions of IL-23 in the normal control group, and the immunoreactivity of IL-23 in the ruptured group was much higher than that in the non-ruptured group (*p* < 0.001). Besides, strong positive expressions can be found around the small vessels and the infiltrated inflammatory cells which were shown in Fig. 2b.

**Table 3:** Immunoreactivity scores of IVD tissues

Cellularity score	Normal control (*n* = 64)	Non-ruptured group (*n* = 104)	Ruptured group (*n* = 128)
0	56	59	32
1	8^a^	45^b^	96^c^

*IVD* intervertebral disc, *0* no labeled cells, *1* at least one positively labeled cell

^a^ From two patients

^b^ From seven patients

^c^ From 14 patients

### RT-PCR results

**Fig. 3:**
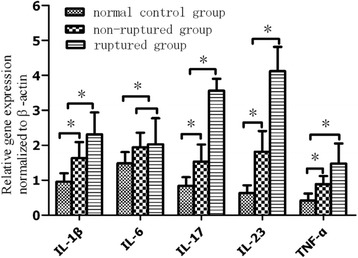
Gene expression of several cytokines normalized to β-actin. The results showed that the mRNA levels of IL-23, IL-17, IL-1β, and TNF-α were significantly higher in the ruptured group when compared to the non-ruptured group except IL-6, and all above cytokines are least detected in the normal control group. *p < 0.05

In order to investigate the gene expression of IL-23 and other inflammatory cytokines, we performed RT-PCR. The results showed that the mRNA levels of IL-23, IL-17, IL-1β, and TNF-α were significantly higher in the ruptured group when compared to the non-ruptured group, but no expression differences were observed at IL-6 between the two groups, and all above cytokines are least detected in the normal control group (Fig. 3). The association degree of the gene expression between IL-23 and IL-17 was calculated by using Pearson correlation coefficient, and significant positive correlations were observed between them (*r* = 0.794, *p* < 0.01).

## Discussion

IL-23 belongs to the IL12 cytokine family and is composed of the unique IL23p19 subunit and the common IL12p40 subunit which is shared with IL-12. Since Oppmann et al. [37] first reported this new cytokine in the journal of *Immunity* in 2000, many articles have focused on its biological functions and its potential therapeutic effects in immunorelated diseases. Because of its extensive biological effect in infections, inflammation, autoimmunity, and tumor, IL-23 has caused much attention in many fields in the last few years. In the current study, we found that IL-23 was expressed in IVD tissues by using the method of RT-PCR and immunohistological staining and found that it is much higher in the ruptured group than that in the non-ruptured group.

As macrophages can secrete IL-23 and they are the mainly infiltrated cells in the herniated IVD tissues [19, 38, 39], we may deduce that IL-23 in the IVD tissues is mainly from the secretion of macrophages. As previous studies [19] indicated that more macrophages were infiltrated in the ruptured group than that in the non-ruptured group, this may be one of the reasons why there were more IL-23 expressed in the ruptured group. However, we also noted that some chondrocyte-like cells were also stained positively with IL-23 antibody. This suggests that NP cells may be another source of IL-23. To determine which kind of cells is the primary source, a further study is needed.

When observing the slides, we also found some interesting phenomenon: more degenerative signs, vessels, and infiltrated cells were found in the ruptured group than that in the non-ruptured group. Meanwhile, significant positive results of IL-23 were found around the small blood vessels and in the area with many infiltrated inflammatory cells. So we may speculate that IL-23 may contribute to the deterioration of IVD by facilitating the neovascularization and strengthening the autoimmune reactions. But the specific mechanisms still need to be further studied.

A previous study by Shamji et al. [9] indicated that the dorsal root ganglion (DRG) treated with NP material failed to show an enhancement of IL-23 staining compared with the sham animals, which seems in contrast to the claims that IL-23 may be contributing to pathological changes in lumbar disc herniation. However, in our work, we directly examined the expression of IL-23 in human nucleus pulposus, not using animal tissues or staining on the DRG. The species variation and the auto-antigenic characterization of the nucleus pulposus may explain this point.

IL-17 is a pro-inflammatory cytokine which is initially discovered to be secreted by Th17 cells and has been found to play an important role in many inflammation- and autoimmune-related diseases. It can work alone or synergistically with other pro-inflammatory cytokines, especially IL-1β, IL-6, and TNF-α to increase the production of diverse mediators of inflammation. Recently, studies have indicated that it is also expressed in human IVD tissues and may play a critical role in the pathogenesis of LDH [33–36]. Cheng et al. [33] reported that Th17 lymphocytes and IL-17 concentrations are higher in the ruptured than those in the non-ruptured discs and are correlated with pain intensity; this suggests that immune activation is responsible, at least in part, for the pain experienced by patients with LDH.

In the current study, we also found that IL-17 was expressed in IVD tissues and its gene expression was much higher in the ruptured group than that in the non-ruptured group, which is consistent with the former investigations. Besides, we also found that there is a higher expression of IL-1β, IL-6, and TNF-α in IVD tissues from LDH than normal controls, especially in the ruptured group, although there is no difference at IL-6 between the ruptured and non-ruptured groups. We also found that there is a positive correlation between IL-17 and IL-23 gene expression. Since studies have proven that IL-23 is essential for differentiation and maintenance of TH17 cells [40], and IL23/IL-17 axis has been found to be associated with many inflammation related diseases [41–44], it can be inferred that this pathway together with other inflammation mediators such as IL-1β, IL-6, and TNF-α may play an important role in the process of LDH.

While the results of this study are encouraging in identifying the expression of IL-23 in IVD tissues, there are some limitations to the report that need to be considered. The included subjects in the study are relatively small, especially in the normal control group. Although infiltrated macrophages seem to be the major source of IL-23, we cannot deny the possibility that it may be secreted by other cells in the disc, so a further study is required to give a definite answer. In addition, we did not study the expression of other cytokines such as IL-22 and MMP-9, which have been reported to be related with IL-23 signaling [45, 46] and may have an important role in the process of LDH.

## Conclusions

Our study demonstrated that IL-23 was expressed in IVD tissues, and it was much higher in the ruptured group than that in the non-ruptured group. In light of the previous and current study on IL-17 and IL-23, we may speculate that the canonical inflammatory related signaling IL-23/IL-17 axis may play a critical role in LDH and further study on the specific mechanisms may provide us a new concept in the therapeutic strategies of LDH.

### Ethics approval and consent to participate

The study was approved by the institutional ethics review board of Tianjin Hospital, and written informed consent was obtained from each patient.

## Abbreviations

HE: hematoxylin and eosin
IVDs: intervertebral discs
LDH: lumbar disc herniation
NP: nucleus pulposus
NR group: non-ruptured group
R group: ruptured group
